# Nutrient sources differ in the fertilised eggs of two divergent broiler lines selected for meat ultimate pH

**DOI:** 10.1038/s41598-022-09509-x

**Published:** 2022-04-01

**Authors:** Angélique Petit, Sophie Réhault-Godbert, Lydie Nadal-Desbarats, Estelle Cailleau-Audouin, Pascal Chartrin, Emilie Raynaud, Justine Jimenez, Sophie Tesseraud, Cécile Berri, Elisabeth Le Bihan-Duval, Sonia Métayer-Coustard

**Affiliations:** 1 INRAE, Université de Tours, BOA, 37380 Nouzilly, France; 2 INSERM, Université de Tours, iBrain, 37000 Tours, France

**Keywords:** Molecular biology, Physiology

## Abstract

The pHu+ and pHu− lines, which were selected based on the ultimate pH (pHu) of the breast muscle, represent a unique model to study the genetic and physiological controls of muscle energy store in relation with meat quality in chicken. Indeed, pHu+ and pHu− chicks show differences in protein and energy metabolism soon after hatching, associated with a different ability to use energy sources in the muscle. The present study aimed to assess the extent to which the nutritional environment of the embryo might contribute to the metabolic differences observed between the two lines at hatching. Just before incubation (E0), the egg yolk of pHu+ exhibited a higher lipid percentage compared to the pHu− line (32.9% vs. 27.7%). Although ^1^H-NMR spectroscopy showed clear changes in egg yolk composition between E0 and E10, there was no line effect. In contrast, ^1^H-NMR analysis performed on amniotic fluid at embryonic day 10 (E10) clearly discriminated the two lines. The amniotic fluid of pHu+ was richer in leucine, isoleucine, 2-oxoisocaproate, citrate and glucose, while choline and inosine were more abundant in the pHu− line. Our results highlight quantitative and qualitative differences in metabolites and nutrients potentially available to developing embryos, which could contribute to metabolic and developmental differences observed after hatching between the pHu+ and pHu− lines.

## Introduction

In chicken, the ultimate pH (pHu) of meat is a key determinant for poultry meat quality and is largely determined by the muscle glycogen content at slaughter^1,2^. The level of glycogen stored in breast muscle, estimated by the glycolytic potential at slaughter, has been shown to be highly heritable (h^2^ = 0.43) and negatively correlated with ultimate pH (rg = − 0.97)^1^. On this basis, two broiler lines called pHu− and pHu+ were divergently selected, from the same population, based on the pHu measurement of the breast muscle^3^. These lines constitute an original model to study the genetic and physiological control of meat quality characteristics associated with muscle glycogen and meat pH variations in chicken.

The pHu+ and pHu− lines were first characterised at slaughter age. At 6 weeks of age, pHu− chickens exhibited a higher breast muscle glycogen content compared to pHu+^3^. This difference was associated with specific blood and muscle metabolic profiles, indicating greater protein catabolism and lipid oxidation in the pHu+ line and greater activation of the glycolytic pathway in the pHu− line^4^. Recently, it has been shown that the divergent metabolic pattern of pHu+ and pHu− lines is set in early during development since, at hatching, the two lines already show differences in protein and energy metabolism that could be related to a different ability to use energy sources at the muscle level^5^.

**Figure 1:**
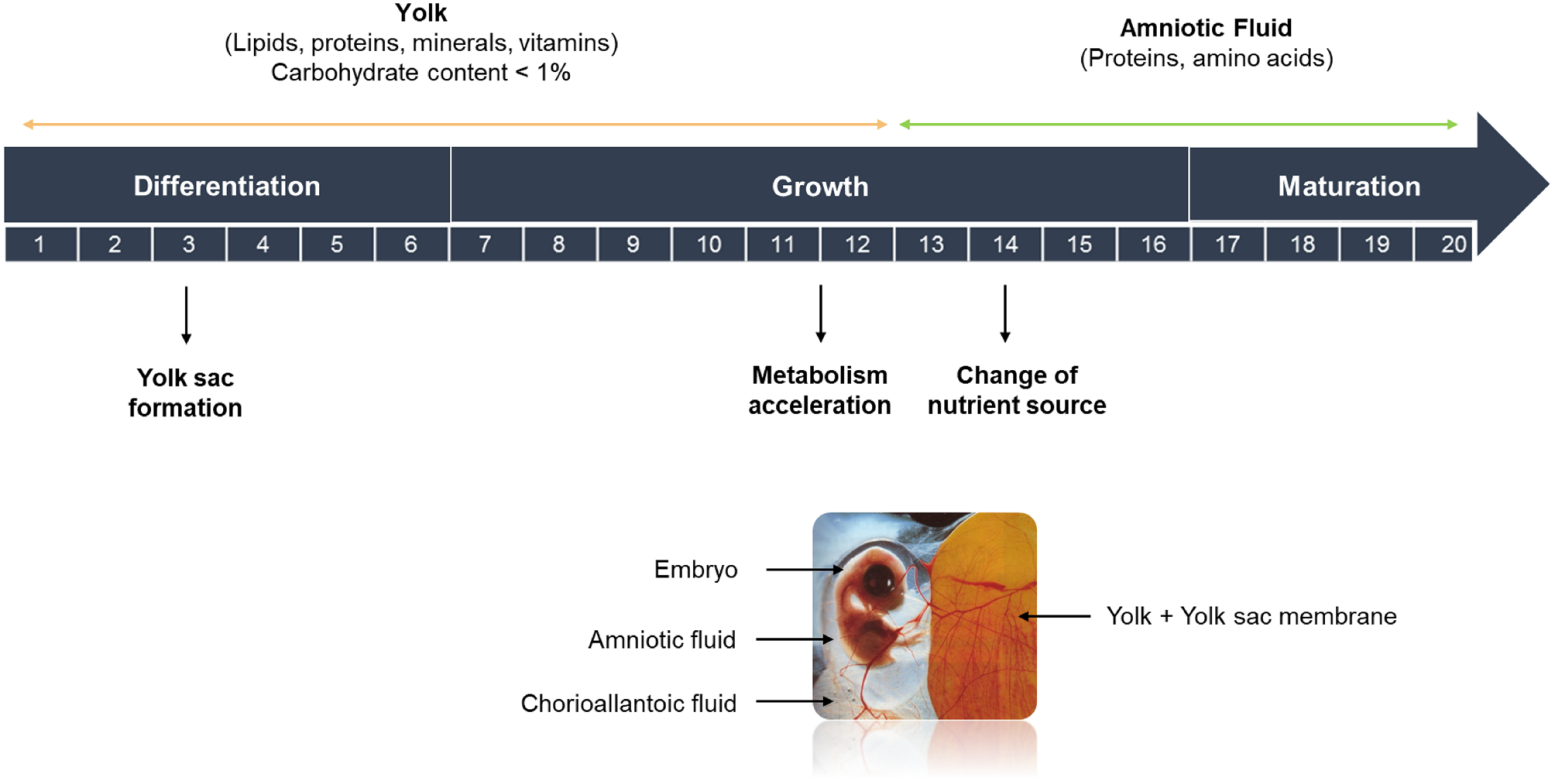
Diagram illustrating the main sources of nutrients for the embryo, the main changes occurring during embryo development and the different embryonic compartments.

The chicken embryo develops in an egg that forms a natural closed chamber containing all the molecules necessary for its survival and development during the 21 days of incubation. The egg is composed of three main extra-embryonic parts that perform vital functions for the embryo, namely the yolk sac, the amniotic sac and the allantoic sac. Within the first week of embryonic development, the yolk sac begins to form from the developing midgut of the embryo intestine (Fig. 1). It contains the yolk, which is composed of 33% fat, 17% protein and traces of carbohydrates and minerals and will be the main source of nutrients for the embryo during incubation^6,7^. During incubation, yolk nutrients are pre-digested by enzymes such as aminopeptidase N (APN) or sucrase isomaltase (SI) to facilitate their uptake and transport to the embryo via the yolk sac membrane (YSM) endothelial cells^8–11^. This transcytosis from the yolk to the embryonic vessels requires the expression in the YSM of amino acid and peptide transporters (*EAAT*, *gLAT2*, *LAT1*, *CAT1* and *PepT1*) and carbohydrate transporters (*SGLT1*, *SLC2A2* and *SLC2A5*)^8,9^.

Around day 12 of development, the metabolic activity of the embryo increases as egg white proteins pass into the amniotic fluid, presumably via the sero-amniotic connection^12^, to supply amino acids to the embryo. These new nutrients in the amniotic sac are consumed via the gastro-intestinal tract^13,14^ and contribute to increasing the body’s glycogen stores^15^.

Since differences in muscle glycogen are already present at hatching between the pHu+ and pHu− lines, the question arises as to whether differences in the nutrient profiles of the yolk and amniotic fluid may contribute to this and interact with the metabolic capabilities of each line. To address this question, the physico-chemical and metabolomic characteristics of the yolk and amniotic fluid were determined at different stages of incubation in the pHu+ and pHu− lines. To further explore the line-related differences, the gene expression pattern of nutrient transporters and digestive enzymes were also determined in the yolk sac membrane.

## Results

### Physico-chemical characteristics of pHu+ and pHu− embryonated eggs

At E0 (before incubation), egg weights were higher in pHu+ than in pHu− (Table 1). This difference was probably due to the higher albumen weight, since yolk and shell weights did not differ between the two lines. Eggs from the pHu+ line had lower proportions of shell, associated with lower static stiffness and breaking strength, and yolk compared to pHu−. Despite a similar weight and pH of yolk (i.e. the main source of nutrients during the first week of embryo development), the embryo weight was higher in pHu+ at E10 (Table 1). At E14, amniotic fluid pH was lower in pHu− than in pHu+.

**Table 1:** Egg characteristics of the pHu+ and pHu− lines (N = 15).

	pHu+	pHu−	P-value
Egg weight (g)	57.88 ± 0.32	54.73 ± 0.18	** < 0.0001**
Storage weight loss (%/day)	0.11 ± 0.01	0.09 ± 0.004	**0.02**
Eggshell weight (g)	5.18 ± 0.14	5.35 ± 0.09	0.36
Eggshell percentage (% EW)	8.89 ± 0.24	9.74 ± 0.22	**0.01**
Static stiffness, Sd (N/mm)	158.62 ± 5.93	174.96 ± 5.82	**0.05**
Breaking strength, F (N)	33.26 ± 1.30	36.49 ± 1.04	0.06
Yolk weight (g)	14.97 ± 0.20	14.87 ± 0.20	0.84
Yolk (% EW)	25.86 ± 0.33	27.17 ± 0.33	**0.008**
Yolk pH (E0)	5.81 ± 0.02	5.80 ± 0.02	0.95
Yolk pH (E10)	7.10 ± 0.07	7.09 ± 0.04	0.90
Albumen weight (g)	31.60 ± 0.52	29.53 ± 0.32	**0.003**
Albumen (% EW)	54.60 ± 0.87	54.42 ± 0.76	0.87
Albumen pH (E0)	9.17 ± 0.02	9.18 ± 0.02	0.77
Amniotic liquid pH (E10)	7.35 ± 0.05	7.36 ± 0.05	0.95
Amniotic liquid pH (E14)	6.95 ± 0.13	6.69 ± 0.05	**0.03**
Embryo weight (g) (E10)	2.95 ± 0.04	2.78 ± 0.06	**0.03**

Data are presented as mean ± s.e.m. Mean comparisons were analysed by Student’s t-test or the Mann–Whitney test. When not specified, values concern egg characteristics before incubation (E0). EW: egg weight.

Significant values are in bold.

### Yolk lipid content and fatty acid composition in pHu− and pHu+

Yolk composition was measured at E0 and E10 in the two lines (Table 2). At E0, the lipid percentage of yolk was higher (32.9%) in pHu+ than in pHu− (27.7%). It decreased between E0 and E10 in both lines, but to a greater extent in pHu+ (− 28.5%) than in pHu− (− 22.3%), so that the percentage of lipid was not significantly different between the two lines at E10. Overall, the fatty acid composition was quite similar between the two lines, except for the percentage of adrenic acid (C22:4 n-6), which was higher in pHu− at E0, and myristoleic acid (C14:1), a marker of polyunsaturated fatty acid oxidation, which was higher in pHu+ than in pHu− at E10.

**Table 2:** Yolk lipid content (%) and fatty acid composition (expressed in % of total lipids) analysis just prior to (E0) and at day 10 (E10) of incubation in pHu+ and pHu− lines (N = 10).

	E0_ pHu+	E0_ pHu−	E10_ pHu+	E10_ pHu−	P-value
Total lipids	32.94 ± 0.92^a^	27.71 ± 1.08^b^	23.56 ± 2.25^bc^	21.53 ± 1.80^c^	**0.0005**
C14	0.33 ± 0.01	0.32 ± 0.006	0.35 ± 0.02	0.34 ± 0.01	0.36
C14:1	0.08 ± 0.003^ab^	0.07 ± 0.006^ab^	0.09 ± 0.006^a^	0.07 ± 0.008^b^	**0.03**
C16	24.97 ± 0.05	24.87 ± 0.18	25.49 ± 0.24	25.11 ± 0.28	0.27
C16:1	2.74 ± 0.07	2.58 ± 0.10	2.99 ± 0.15	2.74 ± 0.17	0.15
C18	7.33 ± 0.14	7.54 ± 0.17	7.39 ± 0.16	7.48 ± 0.21	0.83
C18:1	39.72 ± 0.41	39.54 ± 0.40	38.71 ± 0.49	39.11 ± 0.67	0.46
C18:2 n-6	20.77 ± 0.41	20.86 ± 0.35	21.06 ± 0.31	21.63 ± 0.38	0.40
C18:3 n-3	1.03 ± 0.05	1.02 ± 0.03	1.03 ± 0.04	1.04 ± 0.05	0.83
C20	0.02 ± 0,001	0.03 ± 0.006	0.03 ± 0.002	0.03 ± 0.003	0.43
C20:1	0.21 ± 0.009	0.21 ± 0.007	0.19 ± 0.007	0.21 ± 0.007	0.41
C20:4 n-6	1.62 ± 0.06	1.64 ± 0.07	1.55 ± 0.07	1.56 ± 0.09	0.77
C22:4 n-6	0.25 ± 0.02^b^	0.33 ± 0.02^a^	0.27 ± 0.02^ab^	0.26 ± 0.009^b^	**0.01**
C22:5 n-3	0.17 ± 0.01	0.19 ± 0.01	0.16 ± 0.01	0.15 ± 0.01	0.28
C22:6 n-3	0.78 ± 0.04	0.81 ± 0.05	0.70 ± 0.03	0.73 ± 0.05	0.21
SFA	32.65 ± 0.16	32.76 ± 0.30	33.25 ± 0.23	32.95 ± 0.27	0.31
MUFA	42.74 ± 0.42	42.40 ± 0.39	41.98 ± 0.39	42.12 ± 0.64	0.65
PUFA	24.62 ± 0.42	24.85 ± 0.40	24.77 ± 0.34	24.92 ± 0.68	0.97
n-6	22.64 ± 0.40	22,83 ± 0.36	22.88 ± 0.31	22.99 ± 0.60	0.95
n-3	1.98 ± 0.04	2.02 ± 0.06	1.89 ± 0.04	1.93 ± 0.08	0.42
n-6/n-3	11.47 ± 0.22	11.40 ± 0.27	12.13 ± 0.17	12.00 ± 0.25	0.06

Data are presented as mean ± s.e.m. Mean values without a common letter (a, b, c) differ between groups. Mean comparisons were analysed by ANOVA or the Kruskal–Wallis test. FA: fatty acids; SFA: saturated fatty acids; MUFA: monounsaturated fatty acids; PUFA: polyunsaturated fatty acid.

Significant values are in bold.

To understand the line-related differences in yolk lipid percentage at E0, the expression of genes involved in lipid metabolism was measured in the liver of pHu+ and pHu− breeders, the main site of the de novo synthesis of lipids. The expression of the main enzymes involved in lipogenesis (*FASN*, *FADS1*, *SCD*) was surprisingly over-expressed in the pHu− line (data not shown).

### ^1^H-NMR metabolic profile of pHu− and pHu+ egg yolk aqueous phase

**Figure 2:**
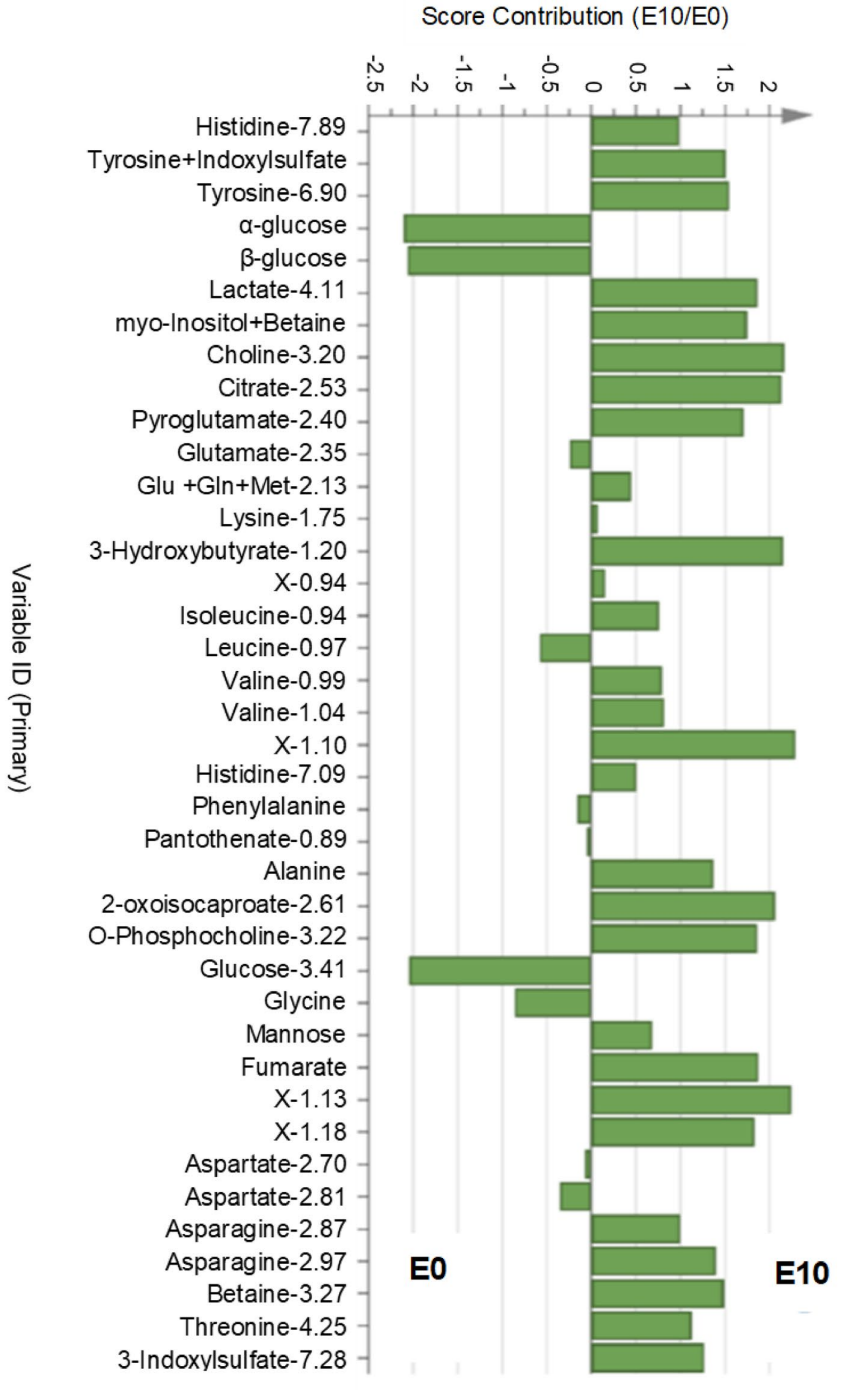
Contribution plot showing the discriminant metabolites identified in the PLS-DA in the yolk between E0 and E10. Glu: glutamic acid, Gln: glutamine, Met: methionine, X-…: unknown compound.

The metabolic profile of the yolk aqueous phase was analysed by ^1^H-NMR spectroscopy at E0 to obtain a snapshot of the initial yolk composition in both lines and at E10 to appreciate the evolution of this composition during embryo development due to nutrient utilisation and enzymatic pre-digestion. Metabolites identified in egg yolk are composed of amino acids and derivatives (leucine, isoleucine, alanine, valine, phenylalanine, methionine, lysine, glutamine, glycine, histidine, tyrosine, threonine, glutamate, pyroglutamate, aspartate, asparagine, betaine, 2-oxoisocaproate and indoxylsulphate, which results from the degradation of tryptophan), sugars and derivatives (glucose, mannose, myoinositol), fatty acid-associated metabolites (choline, 3-hydroxybutyrate), energy-associated metabolites (fumarate, citrate, lactate), and pantothenate, also called vitamin B5 (Fig. 2). A principal component analysis including both lines and based on the set of detected metabolites allowed us to perfectly discriminate egg yolks collected at E0 from those collected at E10, indicating a very different composition between these two stages (Supplementary Fig. 1). The unsupervised analysis showed that the main difference between samples was due to the embryonic day (E0 vs. E10). Supervised partial least square discriminant analysis (PLS-DA) allowed for identifying metabolites in which the concentration evolved between the two stages and allowed us to discriminate them (Fig. 2). Among them, glucose, one of the main energy sources of the developing embryo, decreased the most between E0 and E10. The abundance of some amino acids, such as glycine and leucine, also decreased but to a lesser extent. The PLS-DA also revealed that the abundance of many metabolites increased in the yolk between E0 and E10, regardless of the line. This was the case for amino acids like histidine, tyrosine, isoleucine, valine, alanine, asparagine and threonine, and derivatives like pyroglutamate, 2-oxoisocaproate and 3-indoxylsulphate. Energy-associated metabolites such as lactate, citrate, 3-hydroxybutyrate, mannose and fumarate, or donors of methyl groups, such as choline and betaine, also increased between E0 and E10.

**Figure 3:**
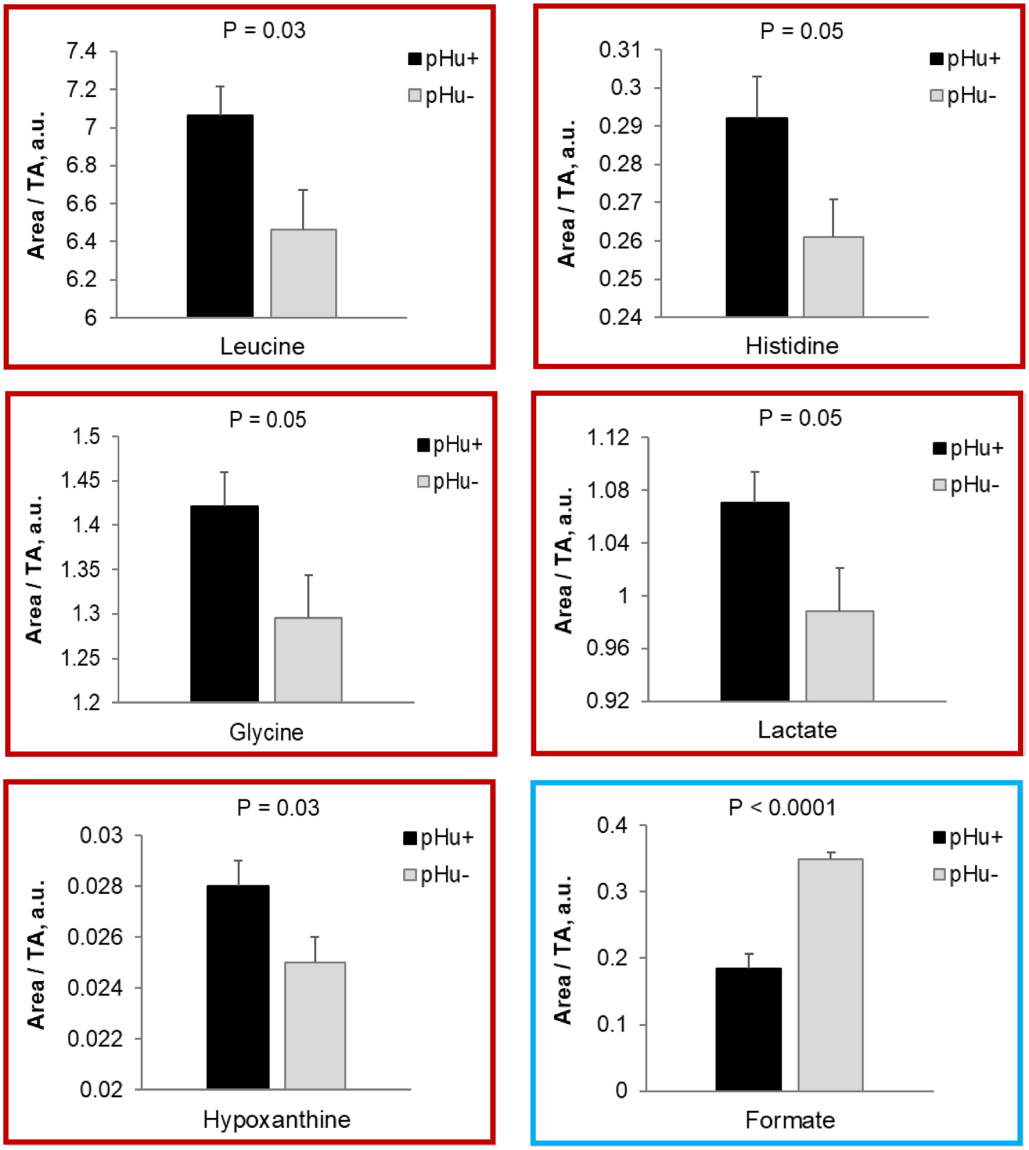
Significantly different metabolites in the yolk of pHu+ and pHu− embryonated eggs at E0 (boxed in red) and E10 (boxed in blue). N = 15. TA: total area, a.u.: arbitrary unit.

However, PLS-DA performed at E0 and E10 on the yolk metabolite profiles of pHu+ and pHu− did not discriminate the two lines, although some metabolites were differentially abundant between them at either stage. Thus, concentrations of leucine, histidine, glycine, lactate and hypoxanthine were higher in pHu+ at E0, and formate, an intermediary metabolite in folate-mediated one-carbon metabolism, was lower in pHu+ at E10 (Fig. 3).

### ^1^H-NMR metabolic profile of pHu+ and pHu− amniotic fluid

**Figure 4:**
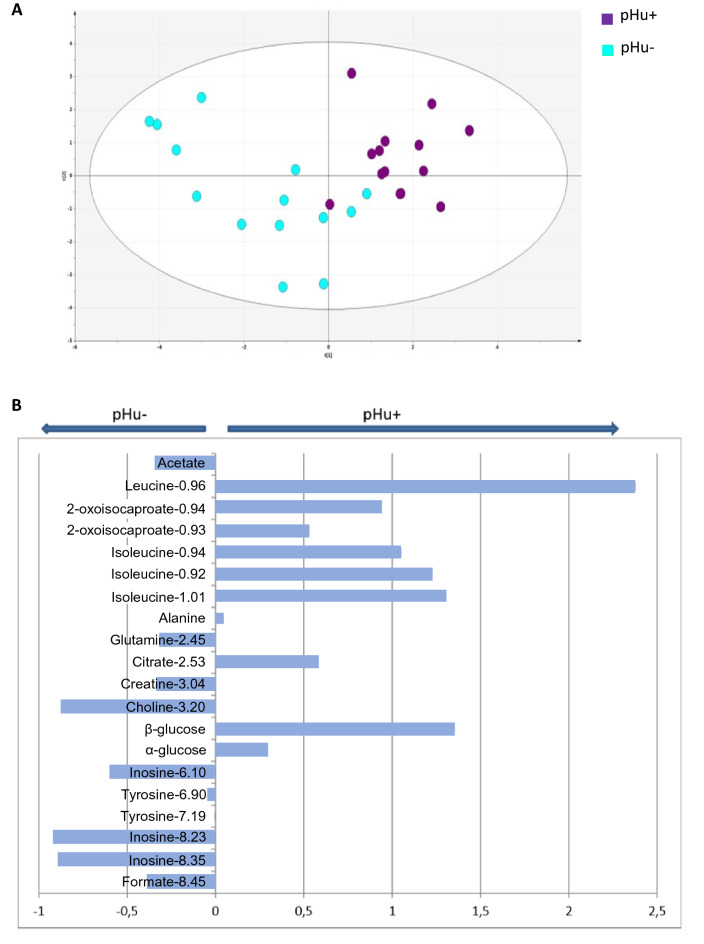
Metabolome score plot following PLS-DA analysis from the ^1^H-NMR spectrum of the amniotic fluid of pHu+ and pHu− eggs (**A**). pHu+ is represented by purple dots and pHu− by blue dots. The descriptive and predictive performance characteristics of the models are R^2^Y = 0.754; Q^2^ = 0.605; CV-ANOVA = 0.00011. Contribution plot giving the discriminant metabolites in the amniotic fluid between pHu+ and pHu− at day 10 of embryonic development (**B**). The horizontal bars represent the enrichment of each metabolite according to the line.

Metabolomics analysis by ^1^H-NMR spectroscopy of the amniotic fluid was performed at 10 days of incubation (E10), i.e., before embryo metabolism intensifies. PLS-DA was carried out on the ^1^H-NMR spectral data and the model obtained had a high cross-validated predictive ability (Q^2^) of 0.605 and an overall proportion of the variation in Y explained by the model (R^2^Y) of 0.754. The reliability of this model was assessed by CV-ANOVA, which gave a P-value equal to 0.00011. The PLS-DA model from the ^1^H-NMR spectrum provided clear discrimination of the pHu+ and pHu− lines (Fig. 4A). The corresponding contribution plot generated by SIMCA+ software was used to identify the metabolites of the amniotic liquid that permitted discrimination between lines. Twenty buckets representing 13 metabolites contributed to this classification (Fig. 4B). Metabolites such as leucine, isoleucine, 2-oxoisocaproate, β-glucose (variable importance in projection (VIP) > 1) and to a lesser extent alanine, citrate and α-glucose were much more abundant in pHu+ line. Conversely, choline and inosine (VIP > 1) and to a lesser extent creatine, glutamine, acetate and formate were more abundant in the pHu− line.

### Gene expression of digestive enzymes and nutrient transporters in the yolk sac membrane of the developing pHu− and pHu+ embryos

The gene expression profiles of digestive enzymes and nutrient transporters were measured in the yolk sac membrane (YSM) at E10, E14 and E17 during embryonic development in both pHu+ and pHu− lines to follow its functionality during the second half of embryonic development.

**Figure 5:**
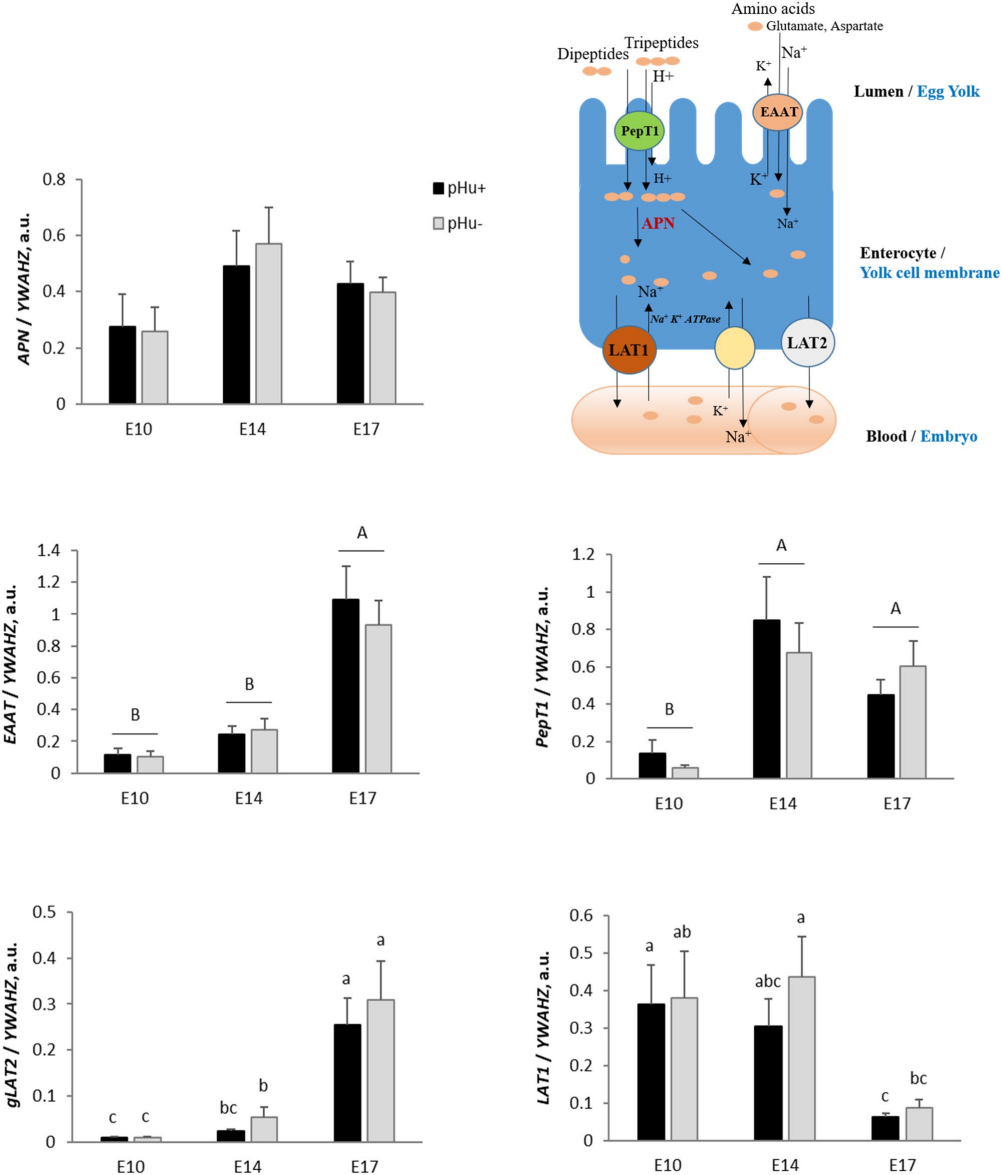
Relative mRNA abundance at E10, E14 and E17 of the digestive enzyme *APN* and peptide and amino acid transporters *EAAT*, *PepT1*, *LAT1* and *gLAT2* in the yolk sac membrane of the pHu+ and pHu− lines. Relative expression of genes (normalised by *YWAHZ* mRNA) was determined by real-time PCR. Data are expressed as means ± s.e.m. (N = 10). Mean comparisons were analysed by the Kruskal–Wallis test. Mean values without a common letter differ between groups (a, b, c) or between ages (**A**,**B**) (P ≤ 0.05). a.u.: arbitrary unit.

The YSM expressed digestive enzymes such as APN, which cleaves neutral and basic amino acids from the N-terminal end of peptides^16^. Its mRNA expression was stable over time (E10 to E17) and was not altered by genetic line (Fig. 5). The expression of several transporters of peptides and amino acids, i.e. *CAT1, EAAT, PepT1, LAT1* and *gLAT2* was determined at the mRNA level (Fig. 5). No line or age effect was evidenced for *CAT1* (data not shown). The expression of *EAAT*, which mediates the absorption of aspartate and glutamate, strongly increased at the end of embryonic development, between E14 and E17, in both the pHu+ and pHu− lines. The expression of *PepT1 (*oligopeptide transporter) increased earlier, between E10 and E14, then remained stable. *gLAT2* expression increased from E10 to E17 in both lines (P < 0.0001), with the main increase occurring between E14 and E17. On the other hand, *LAT1* gene expression was stable between E10 and E14, and then strongly decreased until E17. No line effect was observed on the mRNA expression of any of the studied peptide or amino acid transporters.

**Figure 6:**
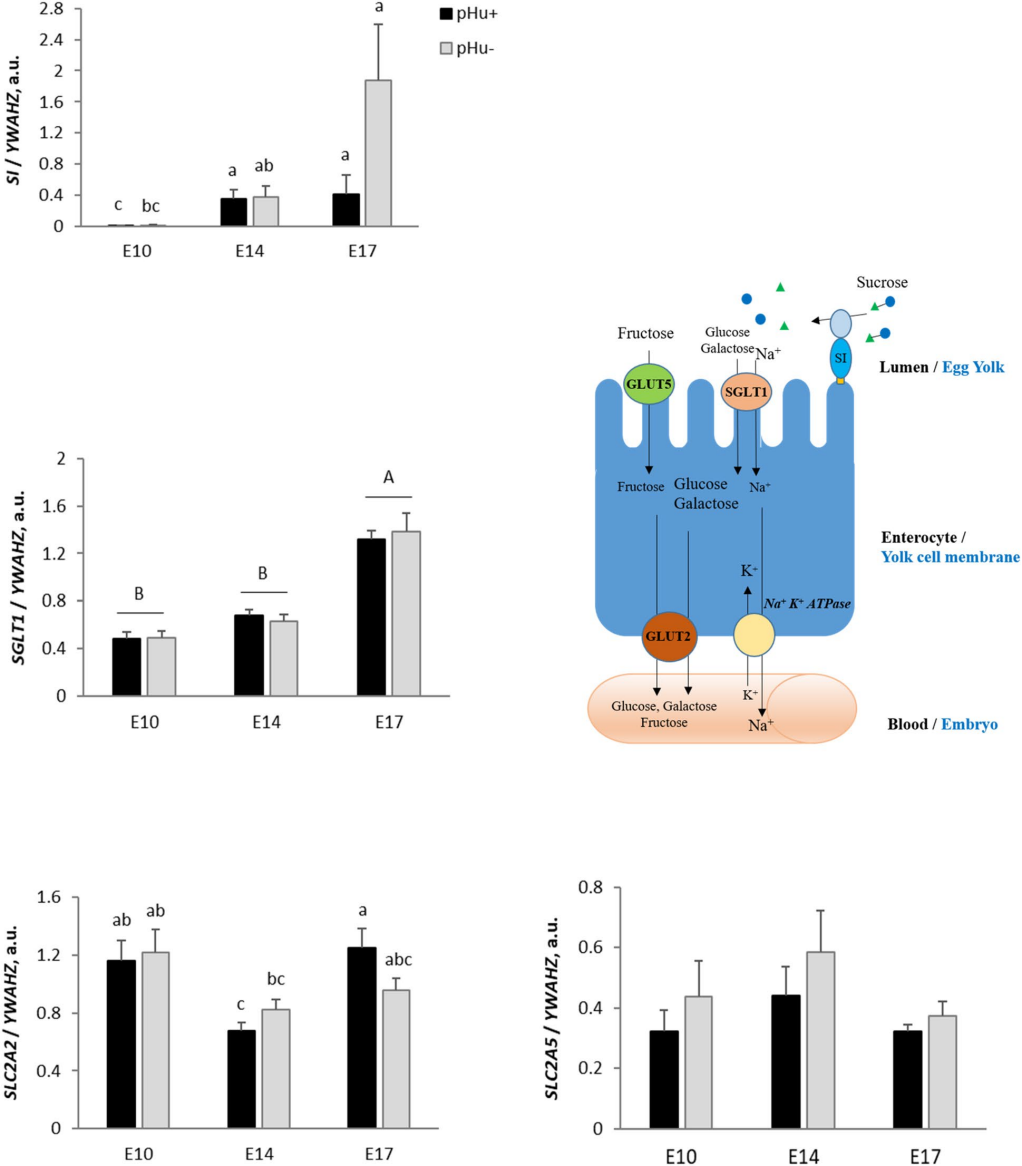
Relative mRNA abundance at E10, E14 and E17 of the digestive enzyme *SI* and carbohydrate transporters *SGLT1, SLC2A2 and SLC2A5* in the yolk sac membrane of the pHu+ and pHu− lines. Relative expression of genes (normalised by *YWAHZ* mRNA) was determined by real-time PCR. Data are expressed as means ± s.e.m. (N = 10). Mean comparisons were analysed by the Kruskal–Wallis test. Mean values without a common letter differ between groups (a, b, c) or between ages (**A**,**B**) (P ≤ 0.05). a.u.: arbitrary unit.

As shown in Fig. 6, the expression of *SI* significantly increased between E10 and E17 in the YSM of both lines (P < 0.01). It is worth noting that the average *SI* expression was multiplied by 4.9-fold between E14 and E17 in the pHu− line, while it barely increased in pHu+. The expression of the sodium and glucose cotransporter *SGLT1* increased between E14 and E17 in both lines (P < 0.05). The expression profile of the glucose, galactose and fructose transporter *SLC2A2* was biphasic in the pHu+ line only. Its expression decreased between E10 and E14 and then increased between E14 and E17, while it remained quite stable in the pHu− line. The expression of the fructose transporter *SLC2A5* did not vary during embryo development. No line effects were observed for the enzyme *SI* and carbohydrate transporters.

### Glycogen content and mRNA expression of genes coding for key glycogenic/gluconeogenic enzymes in the yolk sac membrane

**Figure 7:**
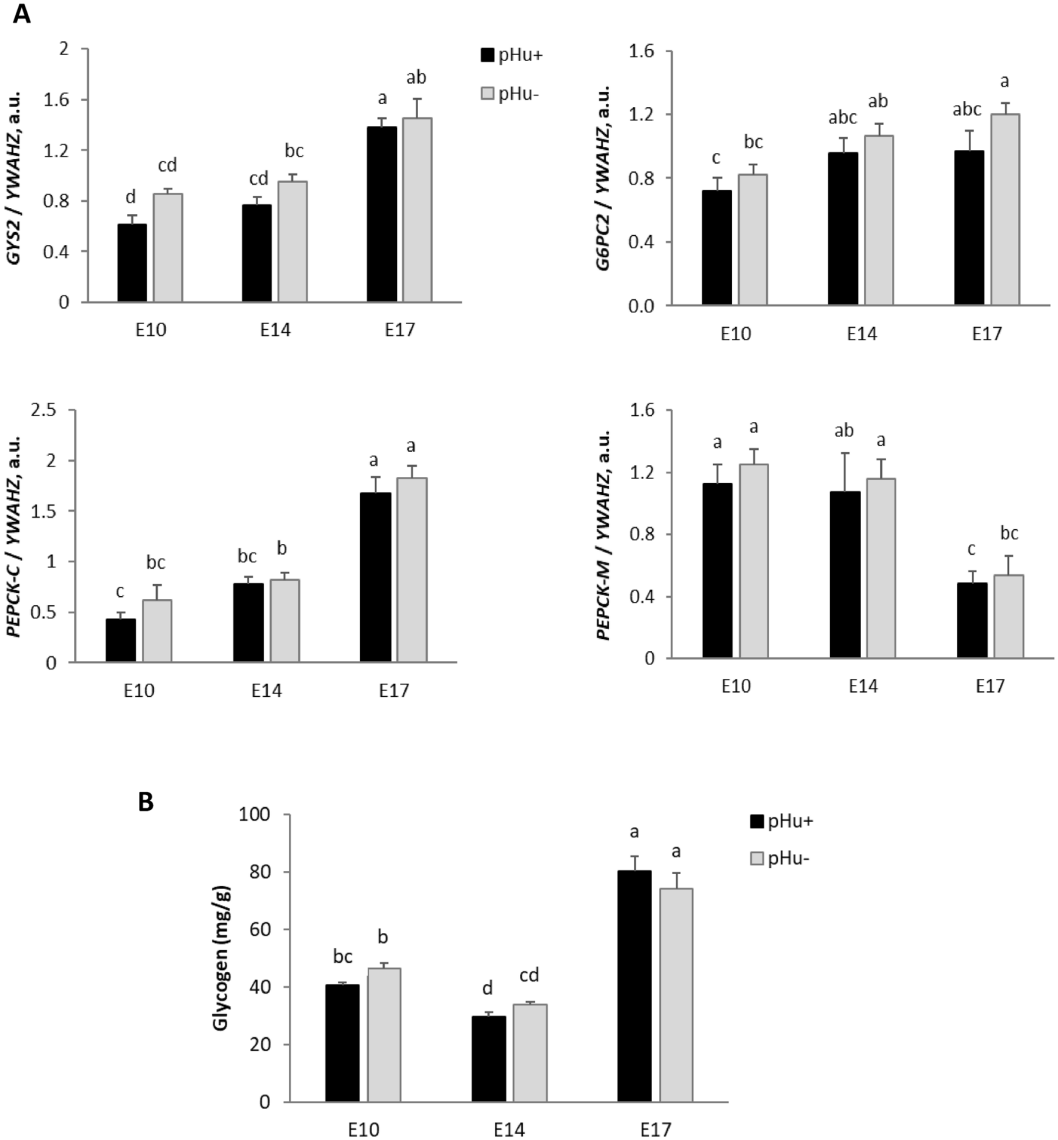
Relative mRNA abundance at E10, E14 and E17 of enzymes involved in carbohydrate metabolism *GYS2*, *G6PC2*, *PEPCK-C* and *PEPCK-M* (**A**) and glycogen measurement (**B**) in the yolk sac membrane of the pHu+ and pHu− lines. Relative expression of genes (normalised by *YWAHZ* mRNA) was determined by real-time PCR. Data are expressed as means ± s.e.m. (N = 10). Mean comparisons were analysed by the Kruskal–Wallis test. Mean values without a common letter differ between groups (a, b, c, d) (P ≤ 0.05). a.u.: arbitrary unit.

The process of glucose utilisation, glycogen storage and/or glucose release in the yolk sac was examined by evaluating the pattern of expression of key enzymes, i.e. *GYS2*, *GK, FBP1, G6PC2*, *PEPCK-C* and *PEPCK-M* during embryonic development (E10, E14 and E17) in the pHu+ and pHu− YSM (Fig. 7A). The mRNA expression of genes coding for *GK* (an enzyme involved in gluconeogenesis or glycolysis) and *FBP1* (an enzyme involved in the conversion of fructose 1,6-biphosphate to fructose 6-phosphate) were not affected by age or genetic line (data not shown). The gene expression of *GYS2*, which catalyses the rate-limiting step in the synthesis of glycogen, regularly increased between E10 and E17 in both lines (P < 0.01). The gene expression of *G6PC2*, which is involved in the conversion of glucose 6-phosphate to free glucose, slightly increased between E10 and E17 in the pHu− line (P < 0.01), but not in pHu+. For *PEPCK*, we considered its two distinct isoforms, i.e. the mitochondrial (*PEPCK-M*) and cytosolic (*PEPCK-C*) forms. *PEPCK-M* and *PEPCK-C* are involved in gluconeogenesis from lactate for *PEPCK-M* and from pyruvate and amino acids for *PEPCK-C*. Gene expression of *PEPCK-M* decreased (P < 0.01) while that of *PEPCK-C* increased (P < 0.001) between E14 and E17 in both the pHu+ and pHu− lines. No line effects were observed for any of the enzymes studied.

The glycogen concentrations in the YSM of pHu− and pHu+ eggs were determined at E10, E14 and E17 (Fig. 7B). The concentration of glycogen decreased significantly between E10 and E14, from 44 to 32 mg/g, then increased sharply between E14 and E17 to reach 77 mg/g on average. There was no line effect on this parameter during embryo development.

## Discussion

Embryo development depends on the integrity of the environment in which it is immersed, but also on the nutrients available. This environment can affect the physiological and morphological development of embryos, with substantial effects on the development of chick phenotypes^17^. At hatching, the divergent lines selected based on breast meat pHu already present distinct phenotypic and metabolic features^5^, which may result from quantitative and qualitative differences in available nutrients *in ovo* and/or their use by the embryo. To test this hypothesis, we first investigated the potential line-related differences in egg characteristics, including the composition of the yolk and amniotic fluid, which are the two main sources of nutrients during egg incubation. Characterising the yolk and amniotic fluid at different time points during incubation also allowed us to follow the kinetics of egg nutrient utilisation by the embryo in these two lines.

The yolk is the main source of lipids and the main source of energy, via fatty acid oxidation, during chick embryonic development^11,15,18^. Before embryo development, a higher percentage of lipids was found in the yolk of pHu+ line (32.9%) than in the yolk of pHu− line (27.7%), with a similar weight of yolk. This could partly explain the higher weight of pHu+ embryos compared to pHu− embryos at E10. The lipid percentage observed in the pHu+ line corresponds to values already reported in broiler breeders^19^ whereas it appears surprisingly low in the pHu− line. The lipid percentage cannot be easily explained by a difference in the hepatic lipid synthesis of breeders from both lines since the main enzymes involved in lipogenesis (FASN, FADS1, SCD) were not over-expressed in the pHu+ line, but surprisingly in the pHu− line. Transport and/or uptake of lipids into the yolk remains to be further explored to explain this difference in lipid deposition in the yolk, knowing that the hens of the two lines received the same diet.

At E10, myristoleic acid (C14:1), a marker of polyunsaturated fatty acid oxidation, was significantly higher in the egg yolk of pHu+ compared to pHu−, which could highlight a more robust lipid oxidation process in this line. The lower eggshell percentage and thus eggshell thickness observed in pHu+ could contribute to greater gas exchange at the shell level, likely to explain the higher level of fatty acid oxidation in this line. Indeed, invagination during early development of the germ leads to chorionic sac and allantoic cavity formation. In turn, their membranes converge at the eggshell^15^. Between embryonic day 5 and 6, the allantoic sac and the chorion fuse to form the chorioallantoic membrane, a highly vascularised membrane involved in oxygen transport^20,21^. Greater access to O_2_ could increase the oxidation of fatty acids, used as a primary source of energy for embryo development^19^, in the pHu+ compared to the pHu− yolk.

Lipid oxidation induces oxidative stress by the production of free radicals^22,23^, which likewise induces an antioxidant response. Among the discriminating metabolites we identified in the yolk at E0, some of them, like histidine, were more abundant in pHu+. The chemical properties of this amino acid are mainly attributed to its imidazole ring, as histidine along with other imidazole-containing compounds present anti-oxidant properties^24^. Therefore, it is likely that greater lipid oxidation observed in pHu+ may induce a greater antioxidant response, which is essential for the protection of the chicken embryo during incubation and of the chick early post-hatch; it thus plays an important role in chick viability^25^. Moreover, glycine, which is involved in both energy production and antioxidant defence through the biosynthesis of creatine and glutathione, respectively^26^, was also found to be much more abundant in the pHu+ than in the pHu− egg yolk at E0. The greater abundance of these two molecules is likely to indicate the response to greater oxidative stress in pHu+, as suggested by the greater amount of hypoxanthine (a marker of oxidative stress, Beauclercq et al.^4^) that was observed at E0 in the yolk of this line compared to pHu−.

Carbohydrates provide energy for the initial phase of embryo development^15,27,28^. The carbohydrate content of the egg is very limited, as its concentration is less than 1% of total nutrients and the percentage of free glucose only about 0.3%^6,29^. Although the carbohydrate content is quite low, glucose is an essential nutrient for energy supply until the chorioallantois has access to the oxygen necessary for fatty acid oxidation^15^. As already described by Yadgary and Uni^30^, yolk glucose decreased between E0 and E10. On the contrary, the mannose content increased significantly between E0 and E10, suggesting that this carbohydrate was not consumed by the embryo during this period. For both glucose and mannose, we did not find differences between lines at these stages. While the concentration of glucose in egg yolk did not vary between the lines, it was much higher at E10 in the amniotic fluid of pHu+ compared to pHu−. Compared to earlier stages, higher expression of some genes related to glucose metabolism was observed in the YSM of both lines during the last third of embryonic development, i.e., after E14. This was the case for SI, whose increased expression in the YSM, classically observed at the end of embryonic development^8^, may reflect increased carbohydrate digestion to provide substrates for monosaccharide transporters such as SGLT1. This increase between E14 and E17, mainly in the pHu− line (P = 0.056), led to an expression level of SI almost 4 times higher in the pHu− line at E17 compared to the pHu+ line. The considerable variability observed at E17, especially in the pHu− line, could mean that the increase in SI mRNA levels occurs more or less early, but could contribute to a greater supply of glucose for pHu− embryos at the end of incubation. Moreover, the expression of G6PC2, involved in the conversion of glucose 6-phosphate into free glucose, also increased between E10 and E17 in the YSM of pHu−, but not in that of pHu+. Except for these line-related differences, no line effect was found for the glycogen content as for the other enzymes involved in glucose utilisation, glucose release and/or glycogen storage in the YSM.

An accumulation of amino acids was observed in the yolk between E0 and E10, regardless of the line. This accumulation is probably due to increased proteolysis following protease activation induced by the pH increase between E0 and E10 (5.80 *vs*. 7.10, respectively). Several proteases and peptidases have already been described in the egg yolk^31^. Cathepsin D, an aspartic protease, is presumed to be a key enzyme for the cleavage of yolk proteins, but other proteases could intervene like aminopeptidase or serine carboxypeptidase. These proteases could cleave the yolk proteins mainly utilised in mid-incubation to supply free amino acids to the developing embryo. Concerning peptide and amino acid transporters in the YSM, their kinetics of expression were similar to those described in the literature^9^. There was no line effect on their expression, which suggested a similar ability to transport amino acids and peptides toward the embryo in the pHu+ and pHu− lines. From E12, the results from literature show changes in the protein profile of the amniotic fluid following protein transfers from the albumen to the amniotic fluid^12,15^. These transfers constitute new sources of available nutrients for the developing embryo during the last week of incubation. Activation of proteases is necessary to generate free amino acids that can be assimilated by the embryo. A study conducted by Da Silva et al.^12^ showed an increase in trypsin-like activities between E12 and E16, and the presence of serine endopeptidases, carboxypeptidases and metalloendopeptidases in the amniotic fluid. The metabolomic analyses conducted in the current study show that leucine was more abundant at E0 in the yolk of pHu+ compared to pHu− embryos and that at E10 both leucine and isoleucine were more abundant in the amniotic fluid of pHu+ than that of pHu− embryos. Leucine and isoleucine are branched-chain amino acids (BCAA) essential for the maintenance and growth of tissues. BCAAs play critical roles in the regulation of energy homeostasis, metabolism, immunity and disease^32–35^. They are not only substrates for protein synthesis, but can also play a role as regulators of intracellular signalling pathways that control cell functions and metabolism, including the protein synthesis process itself. The higher BCAA availability in pHu+ embryos from the amniotic fluid could contribute to explaining the better ability of pHu+ muscles to activate the S6K1/S6 pathway (involved in protein synthesis stimulation) previously shown at hatching, and lead to greater breast muscle growth in this line after hatching^5^. Among the discriminating metabolites identified in the amniotic fluid, 2-oxoisocaproate was also more abundant in pHu+. This compound, also named ketoleucine, is a metabolite of leucine, and may exert with leucine a role in protein and energy metabolism^35^. Whatever the compartment analysed (yolk or amniotic fluid), we observed that the formate concentration was higher in the pHu− line. This can be produced from several substrates (methanol, branched chain fatty acids and amino acids), with some reactions being folate-dependent, others not. The carbon of formate is incorporated into nucleic acids and into the glucogenic amino acid serine. Formate is often considered as a biomarker of the alteration of one-carbon metabolism^36^, and it plays a crucial role in cellular function by providing methyl groups for the synthesis of DNA, polyamine, amino acids, creatine and phospholipids^37^. Because folate-induced one-carbon metabolism is dependent on B-group vitamins as cofactors for many of the key reactions, formate is also often considered as a biomarker of vitamin B deficiency^38^. In addition to being an intracellular metabolite in one-carbon metabolism, formate can also be seen as an interorgan metabolite that distributes one-carbon groups to tissues^36^. The exact significance of the higher presence of formate in the yolk and amniotic fluid of the pHu− line requires further study, but may indicate greater mono-carbon metabolism in this line, potentially related to different nutrient availability compared to pHu+.

The current study provides original results highlighting that selection specifically applied to a muscle criterion (i.e., breast meat pHu, underlying muscle glycogen variations) measured on growing chickens at 6 weeks can lead to correlated changes in the external and internal characteristics of embryonated eggs. This suggests that such selection likely affects hen metabolism related to egg formation and raises the question of the impact of these modifications at the egg level on the subsequent phenotype of the animal (in particular the glycogen content at the muscle level) and their genetic determinism. Transcriptomic and metabolomic analyses previously performed on 6-week-old chickens revealed an intensive use of carbohydrate metabolism to produce energy in the pHu− line and greater solicitation to alternative oxidative pathways (protein catabolism, lipid oxidation) in the pHu+ line^4,39^. In the present study, egg characteristics provided new evidence that the pHu+ embryo would benefit from greater availability of nutrient sources other than carbohydrates, such as lipids from the egg yolk and amino acids from the amniotic fluid, compared with pHu−. Interactions between the *in ovo* nutritional environment and the embryo will have to be further investigated in order to gain a better understanding of the mechanisms underlying the early establishment of muscle phenotypes in the pHu+ and pHu− lines. To this end, the study of steroid and thyroid hormones as well as the vitamin status of the egg could provide additional information. Our results also reveal that the embryonic egg is a putative source of predictive indicators or biomarkers of post-hatching muscle phenotypes, some of which being related to subsequent meat quality. Indeed, the development of myopathies in chicken is often linked to an energy deficit in the muscle^39–41^. Therefore, the pHu+/pHu− model could allow to understand whether the nutritional environment of the embryo can influence the subsequent development of muscle defects in chicken, and to identify *in ovo* markers, whose interest will have to be evaluated in a perspective of application in breeding or selection.

## Conclusion

The present study showed significant differences in egg characteristics and composition between the pHu+ and pHu− lines. The metabolomic profiles of the egg yolk and amniotic fluid highlighted quantitative and qualitative differences in the nutrients potentially available to developing embryos, which could explain subsequent metabolic and developmental differences. Beyond the comparison of the pHu+ and pHu− lines, the characterisation of the different compartments of the egg appears to be a key element to understand the metabolic orientation of the embryos as a function of their genetics and the availability of nutrients before and after hatching. Characterisation of the egg could also contribute to the identification of early biomarkers, accessible *in ovo*, of the animal’s energetic status in relation to its robustness and meat quality.

## Methods

### Egg characterisation and sample collection

All investigators were certified by the French government to handle animal experiments. Our protocol was submitted to the Comité d'Ethique du Val de Loire (CEEA VdL). All experimental protocols were approved by the Comité National de Réflexion Ethique sur l'Expérimentation Animale (number 19). All experiments complied with the ARRIVE guidelines. All methods were carried out in accordance with relevant guidelines and regulations. The study was conducted on eggs and embryo issued from the tenth generation of the two genetic lines that were divergently selected for high or low ultimate pH (respectively called pHu+ and pHu−). They were issued from a commercial broiler line. The experiments were carried out at PEAT INRAE Poultry Experimental Facility (2018, https://doi.org/10.15454/1.5572326250887292E12) (INRAE, Centre Val de Loire, Nouzilly, France). The hens of both groups that produced embryonated eggs received the same diet. Fertile eggs were obtained from laying hens (pHu+ and pHu−) at 28–29 weeks of age, at the peak of lay. Eggs were weighed before and after storage to determine weight loss during storage. Daily weight loss was calculated based on the number of days in storage. Prior to incubation (E0), eggs were weighed. The eggshell quality was evaluated using various measures. The measurements of static stiffness (Sd, quasi-static compression) and breaking strength (F) were carried out using an Instron Type 5543 (Instron, Elancourt, France) on 15 eggs per line. The static stiffness (Sd, N/mm) corresponds to the linear slope of the deformation of the curved force resulting from the load applied to 10 N at a speed of compression of 5 mm/min at the equator of each egg. The breaking strength corresponds to the maximum load (in N) applied to the equator before rupture of the shell. For further egg characterisation, the eggshell, yolk and albumen were separated and weighed (n = 15 per line). The pH of the egg fluids was also measured. The remaining fertile eggs were incubated under standard conditions at 37.8 °C and 56% relative humidity in the experimental hatchery of PEAT to collect the yolk and amniotic fluid on day 10 of incubation (E10) as well as the yolk sac membrane on days 10, 14 and 17 of incubation (E10, E14 and E17) (n = 15 per line and per stage).

### Lipid extraction in the yolk

The total lipid content of the yolk and the percentages of saturated, mono-unsaturated and poly-unsaturated fatty acids (FAs) within the lipid fraction were determined individually in eggs from the pHu+ and pHu− lines at E0 and E10 (n = 10 by line and by stage). Yolk lipid analysis was conducted by trans-methylation with gas chromatography (Perkin Elmer Autosystem, St. Quentin en Yvelines, France) following the protocol described by Chartrin et al.^42^.

### Preparation of ^1^H-NMR samples

Yolk and amniotic fluid were collected by syringe into embryonated eggs from the pHu+ and pHu− lines, at E0 and E10 for the yolk and at E10 for the amniotic fluid (n = 15 per line and per stage). E0 represents the initial stock of nutrients in the yolk and E10 the initial stock of nutrients in the amniotic fluid prior to egg white inflow.

Extraction of yolk metabolites for ^1^H-NMR spectroscopy was carried out according to Wu et al.^43^ using 100 mg of lyophilised yolk. After cold precipitation of lipids and proteins in methanol, samples were homogenised for 30 s prior to the addition of cold chloroform. Samples were homogenised for 1 min, placed on ice for 10 min and then centrifuged (10 min, 12,000*g*, 4 °C). The aqueous phase containing yolk metabolites was collected and dried by solvent evaporation in a SpeedVac (3 h at 35 °C). The dry residue was solubilised in deuterated phosphate buffer (D_2_O-P) containing trimethylsilylpropanoic acid (TSP) at 3.2 mM.

The amniotic fluids were collected at E10 and centrifuged to remove cellular debris (10 min, 3000*g*, 4 °C). The supernatants were ultrafiltered on Amicon columns (cut-off 3 kDa) to remove proteins and peptides of high molecular weight and ultrafiltrates were stored at − 80 °C. Ultrafiltrate samples (150 µL) were prepared in 50 µL of D_2_O-P (pH 7.4) and 10 µL of TSP, used as a reference in NMR spectroscopy. A volume of 80 µL was added to an Eppendorf tube for 16 randomly selected samples to obtain a reference that could be used for normalising the results.

### ^1^H-NMR processing and analysis

The ^1^H spectra were performed on a Bruker 600 MHz cryoprobe spectrometer. The integration of the NMR peaks is proportional to the concentration of metabolites but also to the number of protons that resonate under the peak. The ^1^H-NMR spectra were reduced into consecutive and non-overlapping spectral regions (buckets). Each bucket represents a variable. This allows generating a data matrix that can be analysed by multivariate statistics. Beforehand, steps involving phasing, correction of the baseline and alignment of the chemical shifts were performed. The area of the buckets for the amniotic fluid was normalised according to the total area of the spectrum, whereas for the lyophilised egg yolk, the normalisation was carried out on the reference because of the presence or not of some peaks according to age. Assignments were done using the databases HMDB (http://www.hmdb.ca) and ChenomX NMR suite 8.1 evaluation edition (ChenomX Inc, Edmonton, Canada).

### RNA extraction and RT-qPCR

The messenger ribonucleic acids (mRNAs) were extracted from 100 mg of yolk sac tissue sampled at E10, E14 and E17 using the RNeasy Lipid Tissue Mini Kit (QIAGEN, Courtaboeuf, France) according to the recommendations of the manufacturer. The extracted samples were subjected to DNase treatment (Ambion® DNA-free™, ThermoFisher Scientific, Les Ulis, France). mRNAs were reverse-transcribed using SuperScript™ II Reverse Transcriptase (Invitrogen, Carlsbad, CA, USA) with Random Primers (Promega, Charbonnieres-les-Bains, France). The sequences of forward and reverse primers used to amplify chicken peptide and amino acid transporters (*EAAT, PepT1, LAT1, CAT1, gLAT2),* carbohydrate transporters *(SGLT1, SLC2A2, SLC2A5)*, enzymes (*GYS2, PEPCK-C, PEPCK-M, G6PC2, GK, SI, APN)* and housekeeping gene *YWAHZ* were specifically designed or based on published literature^8,9,16,30,44–46^ (Table 3). The mix containing primers, water and the Takyon (Eurogentec Takyon™ No Rox SYBR^®^ MasterMix dTTP Blue, Angers, France) was distributed in the qPCR plates using ep motion 5070. The cDNA samples were amplified in duplicate by real time PCR using Sybr Green I Master kit (Roche, Mannheim, Germany) and the LightCycler^®^ 480 II apparatus (Roche, Meylan, France). Gene expression levels were estimated on the basis of PCR efficiency and threshold cycle (Ct) deviation of an unknown sample versus a control, as previously described^47^. Their expression was normalised with *YWAHZ* housekeeping gene that was stable between groups.

**Table 3:** Primers used for real-time PCR analysis of chicken embryonic tissues (*Gallus* species).

Gene symbols^a^	Forward	Reverse
*EAAT*	TGC TGC TTT GGA TTC CAG TGT	AGC AAT GAC TGT AGT GCA GAA GTA ATA TAT G
*PepT1*	CCC CTG AGG AGG ATC ACT GTT	CAA AAG AGC AGC AGC AAC GA
*LAT1*	GAT TGC AAC GGG TGA TGT GA	CCC CAC ACC CAC TTT TGT TT
*CAT1*	CAA GAG GAA AAC TCC AGT AAT TGC A	AAG TCG AAG AGG AAG GCC ATA A
*gLAT2*	GCC CTG TCA GTA AAT CAG ACA AGA	TTC AGT TGC ATT GTG TTT GGT T
*GYS2*	CAT CTG TAC ACT GTG CCC ATG TG	TTT GGA GTG ACA ACA TCA GGA TTT
*G6PC2*	CCT TCA CAG ACT GAC ATG GTC ATT A	ATG AGG GAA ATG TGT TGC TAT GAA T
*GK*	TTC GGT GGC TTT TGC ATA ATG	GTC AAA CAC CAT ATG AGC CAT GA
*PEPCK-C*	TGC GAT GGC TCA GAA GAA GA	GAG CCA ACC AGC AGT TCT CAT
*PEPCK-M*	CCG AGC ACA TGC TGA TTT TG	ATG GCC AGG TTG GTT TTC C
*SGLT1*	GCC ATG GCC AGG GCT TA	CAA TAA CCT GAT CTG TGC ACC AGT A
*SLC2A2*	CAC ACT ATG GGC GCA TGC T	ATT GTG CCT GGA GGT GTT GGT
*SLC2A5*	TTG CTG GCT TTG GGT TGT G	GGA GGT TGA GGG CCA AAG TC
*FBP1*	TTC CAT TGG GAC CAT ATT TGG	ACC CGC TGC CAC AAG ATT AC
*SI*	TCA AAT TCC CTA CGA TGT CCA A	AAC AAG AGC CGG TAA CCC AGT A
*APN*	AAT ACG CGC TCG AGA AAA CC	AGC GGG TAC GCC GTG TT
*FASN*	TCT CGA TCT GGC ATA CGA ACT G	CAA CTG GTC CGA GCT TCA AAG
*FADS1*	CAG CAC CAC GCG AAA CC	TCT ACA GAG AGC TTC TTT CCC AAAG
*SCD*	TTT GGC AAT CGG CCG TAT	TGG TAG TTG TGG AAA CCT TCT CCTA
*YWAHZ*	TGA TGT GCT GTC TCT GTT GGA	TGA TAC GCC TGT TGT GAT TGC

Genes^a^ = *EAAT*: excitatory amino acid transporter 3, Na^+^, H^+^ and K^+^ dependent; *PepT1*: oligopeptide transporter; *LAT1*: aromatic amino acid transporter; *CAT1*: Na^+^ independent cationic amino acid transporter; *gLAT2*: Na^+^ independent cationic and Na^+^ dependent neutral amino acid transporter; GK: glucokinase; *GYS2*: glycogen synthase 2; *G6PC2*: glucose-6-phosphatase catalytic subunit 2; *PEPCK-C*: cytosolic phosphoenolpyruvate carboxykinase; *PEPCK-M*: mitochondrial phosphoenolpyruvate carboxykinase; *SGLT1*: Na^+^ dependent glucose and galactose transporter; *SLC2A2*: Na^+^ independent glucose, galactose and fructose transporter; *SLC2A5*: Na^+^ independent fructose transporter; *FBP1*: fructose-1,6-biphosphatase; *SI*: sucrase isomaltase; *APN*: aminopeptidase N; *FASN:* fatty acid synthase; *FADS1*: fatty acid desaturase 1; *SCD:* stearoyl-CoA desaturase; *YWAHZ*: tyrosine 3-monooxygenase/tryptophan 5-monooxygenase activation protein zeta.

Expression of genes involved in the lipid metabolism (*FASN*, *FADS1* and *SCD*) was measured in liver of pHu+ and pHu− breeders. Total liver mRNAs were extracted using the RNA Now protocol (CBX-101, Ozyme); the following steps were the same as those described for the yolk sac (Table 3).

### Quantitative analysis of glycogen

Glycogen measurement was adapted from the protocol described by Monin and Sellier^48^. The YSM (100 mg) was homogenised in 500 µL of H_2_O and 500 µL of cold perchloric acid (PCA, 1 M) using TissueLyser II for 4 min. A volume of 500 µL was centrifuged (20 min, 15,000*g*, 4 °C) to determine the free-glucose concentration (Glucose HK, ref 981779, ThermoFisher Scientific). The remaining volume was suspended with potassium hydroxide (KOH, 5.4 M) and sodium acetate buffer (0.2 M) added with amyloglucosidase (2 mg/mL, Sigma). The samples were incubated for 3 h at 38 °C with stirring (1400 rpm.). Cold PCA (3 M) was added and, after 10 min at 4 °C, samples were centrifuged (15 min, 15,000*g*, 4 °C). Supernatants were collected for total glucose determination (Glucose HK, ref 981779, ThermoFisher Scientific). Scal (ref 981831, ThermoFisher Scientific) and Abtrol (ref 981044, ThermoFisher Scientific) were used for calibration and quality control, respectively.

A range of glycogen was used to determine the regression line y = ax + b.

$$ {\text{Adjusted }}\left[ {\text{Glu HK hydrolysis}} \right]{\text{ in mg/L }} = { 2 }* \, \left[ {\text{Glu HK hydrolysis}} \right] \, *{ 3}.{3 }\left( {\text{dilution factor}} \right) $$

$$ \left[ {\text{Glucose derived from glycogen}} \right]{\text{ in mg/L }} = {\text{ Adjusted }}\left[ {\text{Glu HK hydrolysis}} \right] \, {-} \, \left[ {\text{Glu HK free}} \right] $$

$$ {\text{Amount of glycogen in mg }} = \, (\left[ {\text{Glucose derived from glycogen}} \right]{-}{\text{b}})/{\text{a}} $$

$$ \left[ {{\text{Glycogen}}} \right]{\text{ in mg/g of tissue }} = \frac{{(\left[ {\text{Glucose derived from glycogen}} \right]{-}{\text{b}})/{\text{a}}}}{{{\text{Weight }}\left( {\text{g}} \right)}} $$

### Statistical analysis

Statistical analysis was performed with XLSTAT 2020.5.1 software. Values are presented as means ± standard error of mean (s.e.m.). Results were analysed using ANOVA (n comparisons) or Student’s t-test (two comparisons) after having checked the normality of the residuals distribution (Shapiro–Wilk test) and the variance homogeneity (Levene test for n comparisons and Fisher test for two comparisons). When the residuals were not normally distributed and/or variances were not homogenous between groups, data were analysed with the non-parametric Kruskal–Wallis test (n comparisons) or by the Mann–Whitney test (two comparisons). A level of significance of 5% (p ≤ 0.05) was adopted for all tests.

In ^1^H-NMR spectroscopy, a principal component analysis (PCA) with unsupervised analysis, followed by supervised and discriminant analysis (PLS-DA), were performed using MetaboAnalyst (https://www.metaboanalyst.ca/) and SIMCA software (SIMCA 13.0.3 software (Umetrics, Umeå, Sweden). The overall quality of the model was appreciated by the R^2^Y, defined as the proportion of variance in Y explained by the predictive component of the model and Q^2^ the predictive ability of the model classes. The higher R^2^Y and Q^2^, the better the separation between the pHu+ and pHu− lines or stages. A variance analysis (CV-ANOVA) was then applied to further evaluate the significance of the results. Metabolites included in the model with a variable importance in projection (VIP) greater than 1 were considered as important. Samples out of the confidence index (outliers) were removed from the analysis.

## Supplementary Information

Supplementary Legends.

Supplementary Figure 1.

## Data Availability

The data that support the findings of this study are available from the corresponding author upon reasonable request.
