# Supplementary material for: Nutrient sources differ in the fertilised eggs of two divergent broiler lines selected for meat ultimate pH
Source: Sci Rep. 2022 Apr 1;12:5533. doi: 10.1038/s41598-022-09509-x (PMC8975873; doi:10.1038/s41598-022-09509-x)
Supplement: Supplementary file 1 — Supplementary Legends. [file 41598_2022_9509_MOESM1_ESM.docx]

**Supplemental Figure 1.** Comparison by unsupervised analysis (PCA) between the different sampling times (E0 and E10) in the pHu+ and pHu- lines.
